# Presumed veterinary niclosamide-induced retinal toxicity in a human: a case report

**DOI:** 10.1186/s13256-023-03868-3

**Published:** 2023-03-26

**Authors:** Fatemeh Bazvand, Hamid Riazi-Esfahani, Farhad Salari

**Affiliations:** 1 Eye Research Center, Farabi Eye Hospital, Tehran University of Medical Sciences, Tehran, 1336616351 Iran; 2 Retina & Vitreous Service, Farabi Eye Hospital, Tehran University of Medical Sciences, Tehran, Iran

**Keywords:** Niclosamide, Drug toxicity, Bull’s-eye maculopathy, Retinopathy

## Abstract

**Background:**

To report the first case of bull’s eye maculopathy associated with veterinary niclosamide.

**Case presentation:**

A 27-year-old Iranian female presented with a history of reduced vision and photopsia since 3 years, after accidental ingestion of four boluses of veterinary niclosamide. Fundus examination showed atrophy in parafoveal retinal pigmentary epithelium, appearing as bilateral bull’s-eye maculopathy. Optical coherence tomography revealed disruption of the parafoveal ellipsoid zone and outer retinal thinning, appearing as a flying saucer sign. Electroretinography displayed decreased scotopic and photopic amplitudes with normal waveform in both eyes. The causality score was 4, showing “possible” retinopathy due to niclosamide according to Naranjo’s causality assessment algorithm. Based on clinical and ancillary findings, a diagnosis of niclosamide-induced maculopathy was made.

**Conclusion:**

Veterinary niclosamide is an anthelmintic drug that in higher doses could be detrimental to the human retina. Awareness about its side effects and appropriate drug labeling could prevent accidental toxicity.

## Background

Niclosamide has been commonly prescribed for the treatment of intestinal parasite infections for several decades. This drug kills tapeworms by inhibiting oxidative phosphorylation in the mitochondria and anaerobic adenosine triphosphate (ATP) production [1]. Recent studies have shown its potential in treating many other diseases including Parkinson’s disease, diabetes, viral and microbial infections, and inhibition of myopia progression [2–5]. In addition, other studies have demonstrated the efficacy of niclosamide in the treatment of various cancers [6]. Niclosamide is a well-tolerated drug in humans, which can be used orally at 2 g/day, leading to serum concentrations of 0.25–6.0 µg/mL. This concentration is not toxic and is within the range of active antiviral concentrations in humans [7]. At safe doses, the side effects of niclosamide are mostly mild and transient. Reported adverse drug reactions are gastrointestinal disturbances and skin reactions [8]. Herein, we report the first case of maculopathy associated with overdosing of veterinary niclosamide.

## Case report

A 28-year-old Iranian female was referred to Farabi Eye Hospital complaining of vision loss and photopsia in both eyes for 3 years. Her father was a ranchman, and she accidentally took four boluses of veterinary niclosamide (1250 mg in each bolus) 3 years ago. She announced that the visual problems had begun just a few days after taking the tablets. She was evaluated by a neurologist at that time and received a short course of high-dose oral corticosteroid, with a probable diagnosis of bilateral optic neuritis as a result of veterinary niclosamide [brain magnetic resonance imaging (MRI) and other neurologic workups were normal]. The patient’s adherence to treatment and detailed timing and dosage of corticosteroid treatment was not available. However, she declared that her vision did not improve nor worsen with the treatment. Thereafter, her visual symptoms remained stable over the past 3 years. At the time of presentation to our clinic, she did not have gastrointestinal or neurologic symptoms, and only complained about her vision. Unfortunately, we did not have access to her primary clinical or ophthalmologic examinations including visual acuity and the probable change in visual acuity after corticosteroid treatment. She did not consume any other medications or drugs during this period. Also, she did not consume alcohol and denied regular smoking. She denied nyctalopia, hemeralopia, and photophobia. There was no known parental consanguinity and her family history was negative for vision loss and retinal disorders. Genetic testing was not possible because of the economic situation of the patient. She was an unemployed girl without any pregnancy history. Her past medical history was negative for any other significant medical or psychological problems.

**Fig. 1:**
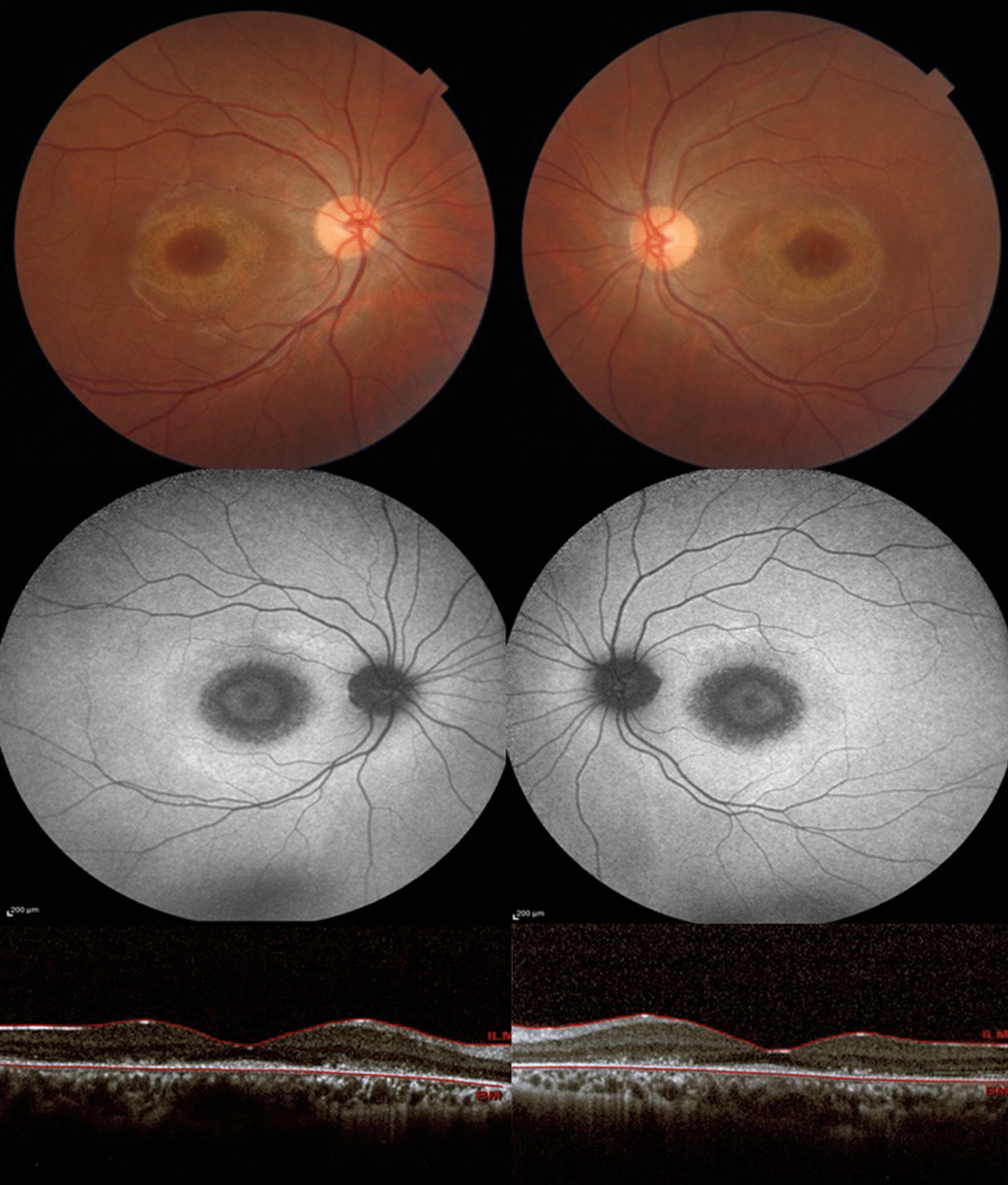
Color fundus photo shows parafoveal pigmentary changes (top images). Blue fundus autofluorescence reveals target-like parafoveal rings consisting of symmetric bull’s-eye maculopathy (middle images). Optical coherence tomography shows outer retina thinning, parafoveal ellipsoid zone loss, and relative sparing of fovea known as early “flying saucer sign” (bottom images)

At the presentation time, physical examinations revealed normal vital signs and a Glasgow Coma Scale (GCS) score of 15. Her neurologic examination was not significant except for reduced visual acuity. On ophthalmologic examination, her best-corrected acuities were 20/50 and 20/63 for the right and left eye, respectively. She had normal color vision and pupillary reaction in both eyes. Anterior segment and vitreous examinations were unremarkable and intraocular pressures were normal. Fundus examination showed pink optic discs with a cup-to-disc ratio of 0.2 and a disclosed atrophy in parafoveal retinal pigmentary epithelium appearing as bilateral bull’s-eye maculopathy (Fig. 1). There were no other significant findings on physical examination.

**Fig. 2:**
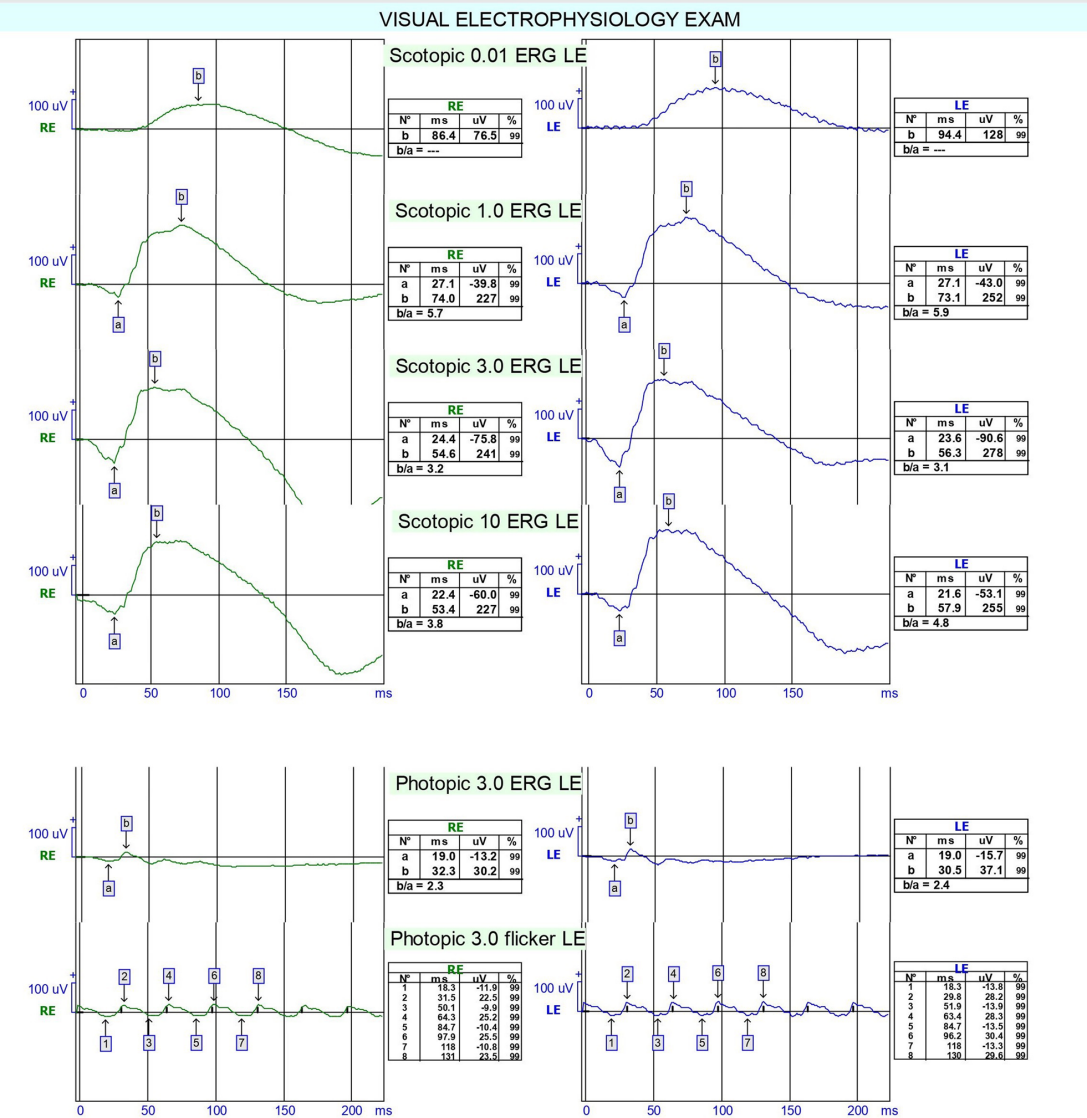
Full-field ERG displaying decreased scotopic and photopic amplitudes with normal waveform in both eyes of the patient

Fundus autofluorescence revealed annular hyper-autofluorescence in the parafoveal region consistent with bull’s-eye maculopathy (Fig. 1). Optical coherence tomography (OCT) (Heidelberg Engineering GmbH, Heidelberg, Germany) showed disruption of the parafoveal ellipsoid zone and outer retinal thinning appearing as a flying saucer sign (Fig. 1). Electroretinography (ERG) displayed decreased scotopic and photopic amplitudes with the normal waveform in both eyes (Fig. 2). Electrooculography disclosed an Arden ratio of 240% in the right eye and 202% in the left eye. On Naranjo’s causality assessment algorithm, the causality score was 4 showing “possible” retinopathy due to niclosamide [9]. Based on her history, clinical examination, and ancillary tests, a diagnosis of niclosamide-induced maculopathy was made. As the diagnostic assessment revealed the damage to the macula was irreversible, we did not consider any treatment option for the patient and only schedule her for follow-up visits.

## Discussion

This case describes a long-standing retinopathy in a 28-year-old woman as a result of veterinary niclosamide toxicity. Although there are several reports of persistent retinopathy due to toxicity from other drugs in the literature, this is the first report of bull’s-eye retinopathy due to niclosamide toxicity.

Niclosamide is a Food and Drug Administration (FDA)-approved drug for treating tapeworms. The main anthelmintic effect is because of the interference with respiration and blocking glucose uptake in targeted cells. Many studies have demonstrated niclosamide as a promising drug against cancer cells. In a study by Li *et al*., niclosamide effectively interferes with the growth and angiogenesis of retinoblastoma. Their molecular study revealed this drug destroys retinoblastoma cells by inhibiting the Wnt/β-catenin pathway [10]. Other proposed mechanisms of action include suppression of STAT3 and NF-ĸB pathways [11]. Moreover, recent publications have shown the potential usage of this drug in the management of COVID-19 [12]. In addition, an animal study by a Liu *et al*. found that niclosamide could effectively inhibit the progression of myopia [5]. Patients consume 2 g for up to 7 days for the treatment of intestinal parasites. At this conventional dosage, it seems to be a safe drug with minimal adverse reactions.

In the literature, human retinopathy due to accidental consumption of veterinary drugs is limited to closantel ingestion. There are several reports of severe visual complications following closantel consumption [13]. To the best of our knowledge, this is the first report of veterinary niclosamide-induced neuroretinal toxicity. Our patient showed a combination of findings including bull’s eye maculopathy, paracentral outer retina and ellipsoid attenuation on OCT, and cone and rod dysfunction on ERG.

Differential diagnoses for this patient include cone dystrophy, Stargardt’s disease, and other drug toxicity. ERG may help in narrowing the differential diagnosis. Regarding the absence of photophobia, nystagmus, poor vision, negative familial history, and unrelated parents, as well as the emergence of symptoms shortly after taking veterinary niclosamide, cone dystrophies and Stargardt’s disease were unlikely. Significant thinning of central foveal thickness, loss of ellipsoid zone in the parafovea, and relative central foveal sparing resemble chloroquine toxicity. Also in the literature, optic neuropathy with bull’s eye maculopathy has been reported as an adverse effect of some drugs such as quinine and chemotherapy with a combination of cytarabine and daunorubicin [14, 15].

Although a lack of nystagmus or photophobia and a negative family history reduce the possibility of inherited retinal disorders, they are still likely. In addition, we could not perform genetic testing for her due to socioeconomic problems, and therefore inherited retinal disorders could not be ruled out. However, our patient seems to have been initially misdiagnosed with optic neuritis. Instead of an ophthalmology consultation, she had been treated with corticosteroids. Retinal toxicity should be kept in mind for future cases and a prompt ophthalmologist is advisable.

The best treatment protocol is unknown. In similar scenarios such as toxicity with cosantel, early treatment with intravenous methylprednisolone 1 g per day and intravenous erythropoietin 10,000 IU twice a day showed efficacy [13]. Another potential treatment in the acute stage is plasmapheresis [16]. As this drug is largely eliminated by the kidneys, hemodialysis may be effective [17].

The possible pathophysiological explanation for the disruption of the ellipsoid zone could be its inhibitory effect on mitochondrial oxidative phosphorylation, which results in severe photoreceptor and neural cell damage. Additionally, niclosamide is known to affect the intracellular pH and lysosome function, and thus inhibiting autophagy of the retinal pigment epithelial (RPE), affecting photoreceptor cell membrane stability function [18]. The possible cause of foveal sparing in our case could be the accumulation of macular pigments in the central fovea, which preserve photoreceptor cells from oxidative damage. This case showed a severe irreversible RPE photoreceptor and ganglion cell toxicity due to niclosamide overdosing.

## Conclusion

Veterinary niclosamide is an anthelmintic drug that, in higher doses, could be detrimental to the human retina. It seems that veterinary niclosamide has a long-term detrimental impact on the mentioned structures, with no discernible recovery after 3 years. Appropriate drug labeling along with awareness about its side effects could prevent accidental toxicity.

## Data Availability

Not applicable.

## Abbreviations

OCT: Optical coherence tomography
ERG: Electroretinography
EOG: Electrooculography
FDA: Food and Drug Administration
